# Inadvertent Arterial Placement of a Peripherally Inserted Central Catheter in an Infant With Dilated Cardiomyopathy: A Case Report

**DOI:** 10.7759/cureus.61053

**Published:** 2024-05-25

**Authors:** Kaoru Tsuboi, Saki Endo, Norihiko Tsuboi, Shunsuke Nosaka, Shotaro Matsumoto

**Affiliations:** 1 Critical Care Medicine, National Center for Child Health and Development, Tokyo, JPN; 2 Radiology, National Center for Child Health and Development, Tokyo, JPN

**Keywords:** pediatrics, never event, peripherally inserted central catheter line insertion, pediatric intensive care unit, interventional ultrasound

## Abstract

Peripherally inserted central catheter (PICC) placement under real-time ultrasound guidance has emerged as a favorable procedure in children as a method to efficiently obtain central access. Nevertheless, small infants with hemodynamic instability are at high risk of complications and extra precautions are necessary. We present a case of an inadvertent arterial placement of a PICC in a two-month-old infant with dilated cardiomyopathy and decompensated heart failure. Differentiation of arteries and veins under ultrasonographic evaluation may sometimes be difficult when the applied tourniquet pressure exceeds the patient’s arterial blood pressure. In particular, arterial flow can be easily compromised by applying tourniquet pressure in small children with low blood pressure. A thorough understanding of the upper extremity vascular anatomy, basic scanning techniques, and meticulous preparation especially in small infants with hemodynamic instability are essential for maintaining the safety and efficacy of this procedure.

## Introduction

Peripherally inserted central catheter (PICC) has emerged as a favorable procedure in children due to its safety and reliability to efficiently obtain central access [1]. Real-time ultrasound guidance in central line placement has demonstrated low rates of complications, shorter procedure times, and higher success rates, and its adoption as standard care has been recommended by several health organizations [2]. Nevertheless, small infants with hemodynamic instability are at high risk of complications and extra precautions are necessary. We present a case of an inadvertent arterial placement of PICC in a two-month-old infant with dilated cardiomyopathy and decompensated heart failure.

## Case presentation

A two-month-old infant weighing 4.7 kg was scheduled for PICC placement. The patient had no significant birth history but presented with signs of severe left ventricular dysfunction and decompensated heart failure and was diagnosed with dilated cardiomyopathy. He had been on mechanical ventilation and catecholamine support for the previous two weeks in the pediatric intensive care unit. Replacement with a PICC was considered due to prolonged indwelling of the central venous line. His blood pressure at the time of the procedure was around 60/40 mmHg. His heart rate was 140 to 150, capillary refill time was three seconds and SpO_2_ was 96% (under invasive mandatory ventilation, F_I_O_2_ 0.4).

**Figure 1:**
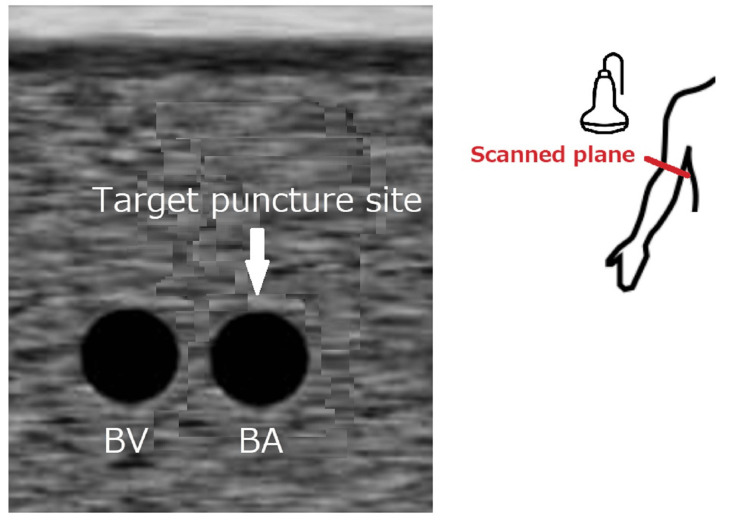
Scheme of the ultrasound image The diameters of the two vessels were nearly identical, and vascular compressibility on manual pressure application was confirmed on both vessels. BA: brachial artery; BV: brachial vein

Real-time ultrasound-guided insertion of PICC was performed. As initial attempts at the cephalic vein failed, a tourniquet was tightly applied around the patient’s shoulder to search for the next target. Two round vessels were identified on a transverse plane on the pre-scan. These were initially assumed to be the brachial artery and vein based on their anatomical locations. However, repeated ultrasonography scans showed absent color flow within the lumen of neither vessel. The diameters of the two vessels were nearly identical, and vascular compressibility as well as reverse color flow on application of manual pressure at the distal portion were confirmed (Figure 1).

**Figure 2:**
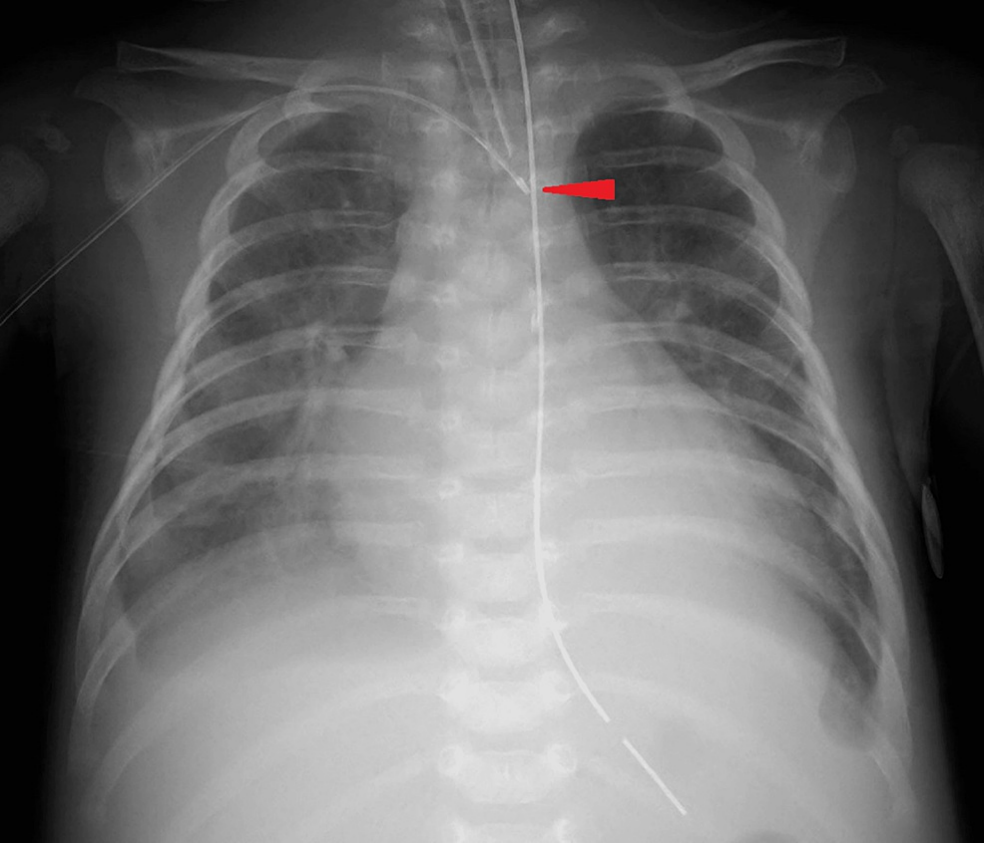
Chest radiography after insertion of a peripherally inserted central catheter The arrowhead shows the tip of the peripherally inserted central catheter at the aorta.

Based on these ultrasound findings, both structures were determined as veins, and the medial vessel was targeted. On puncture, the course of the needle tip was carefully visualized, with the tip maintained in the center of the vascular lumen to avoid posterior wall puncture. Blood return through the advanced needle was dark and non-pulsatile. The procedure was completed without any immediate concerns noted. However, postprocedural chest X-ray showed the catheter tip to be at the left of the midline (Figure 2).

Blood gas obtained from the inserted PICC line revealed P_a_O_2_ of 101.0 mmHg indicating arterial placement. The catheter was immediately removed with no serious consequences.

## Discussion

Inadvertent arterial placement is most likely with the brachial vein due to its proximity to the brachial artery. Known complications of inadvertent arterial puncture include brachial arteriovenous fistula, pseudoaneurysm formation, and the potential for distal arterial flow compromise [1]. In general, arteries can be differentiated from veins by several characteristics, including round transverse images, partial compression on pressure application, pulsatile color flow within the lumen on ultrasonographic evaluation, and bright red pulsatile blood return on puncture. However, none of these indicators are useful when the applied tourniquet pressure exceeds the patient’s arterial blood pressure. Arteries are reported to be compressible in patients with a systolic arterial pressure below 60 mmHg [3]. Although rare, inadvertent arterial PICC placement has been reported predominantly in small infants and neonates [4-5]. This may be due to the low blood pressure and the small diameters of the arteries, where luminal flow can be easily compromised by tourniquet application. Previous reports have least recommended brachial veins for PICC insertion in children due to their potential risk of inadvertent arterial placement and possible damage to the median nerve [1].

In the present case, PICC insertion was attempted on a small infant with a low systolic blood pressure of around 60 mmHg. The applied tourniquet pressure was probably too high, exceeding the patient's arterial pressure leading to misidentification of the artery and inadvertent arterial puncture. The reason why the returned blood on the puncture was dark is unknown but may be explained by the fact that the intima and inner media of the arterial wall receive oxygen primarily from luminal blood flow [6]. Oxygen saturation within the arterial lumen may have decreased due to transportation to the intima and inner medial layers of the artery under prolonged tourniquet application.

A thorough understanding of the upper extremity vascular anatomy, basic scanning techniques, and meticulous preparation especially in small infants with hemodynamic instability are essential for maintaining the safety and efficacy of this procedure.

## Conclusions

Small infants with hemodynamic instability are at high risk of complications when inserting PICC. Classic indicators of arterial placement may not be evident in these circumstances. A thorough understanding of the anatomy as well as meticulous preparation, especially in small children with low blood pressure, are essential for maintaining the safety of this procedure.
